# Comprehensive risk profiles of family history and lifestyle and metabolic risk factors in relation to diabetes: A prospective cohort study

**DOI:** 10.1111/1753-0407.13289

**Published:** 2022-06-28

**Authors:** Chaojie Ye, Yiying Wang, Lijie Kong, Zhiyun Zhao, Mian Li, Yu Xu, Min Xu, Jieli Lu, Shuangyuan Wang, Hong Lin, Yuhong Chen, Weiqing Wang, Guang Ning, Yufang Bi, Tiange Wang

**Affiliations:** ^1^ Department of Endocrine and Metabolic Diseases, Shanghai Institute of Endocrine and Metabolic Diseases, Ruijin Hospital Shanghai Jiao Tong University School of Medicine Shanghai China; ^2^ Shanghai National Clinical Research Center for metabolic Diseases, Key Laboratory for Endocrine and Metabolic Diseases of the National Health Commission of the PR China, Shanghai Key Laboratory for Endocrine Tumor, State Key Laboratory of Medical Genomics, Ruijin Hospital Shanghai Jiao Tong University School of Medicine Shanghai China

**Keywords:** diabetes, family history, lifestyle, metabolic disorder, risk profile, 糖尿病, 家族史, 生活方式, 代谢紊乱, 风险特征

## Abstract

**Background:**

Family history of diabetes, unhealthy lifestyles, and metabolic disorders are individually associated with higher risk of diabetes, but how different combinations of the three risk categories are associated with incident diabetes remains unclear. We aimed to estimate the associations of comprehensive risk profiles of family history and lifestyle and metabolic risk factors with diabetes risk.

**Methods:**

This study included 5290 participants without diabetes at baseline with a mean follow‐up of 4.4 years. Five unhealthy lifestyles and five metabolic disorders were each allocated a score, resulting in an aggregated lifestyle and metabolic risk score ranging from 0 to 5. Eight risk profiles were constructed from combinations of three risk categories: family history of diabetes (yes, no), lifestyle risk (high, low), and metabolic risk (high, low).

**Results:**

Compared with the profile without any risk category, other profiles exhibited incrementally higher risks of diabetes with increasing numbers of categories: the hazard ratio (HR, 95% confidence interval [CI]) for diabetes ranged from 1.34 (1.01–1.79) to 2.33 (1.60–3.39) for profiles with one risk category, ranged from 2.42 (1.45–4.04) to 4.18 (2.42–7.21) for profiles with two risk categories, and was 4.59 (2.85–7.39) for the profile with three risk categories. The associations between the numbers of risk categories and diabetes risk were more prominent in women (*p*_interaction_ = .025) and slightly more prominent in adults <55 years (*p*_interaction_ = .052).

**Conclusions:**

This study delineated associations between comprehensive risk profiles with diabetes risk, with stronger associations observed in women and slightly stronger associations in adults younger than 55 years.

## Graphical abstract

**Highlights**

- Compared with the profile without any risk category, of profiles with one risk category, the profile with family history conferred the highest (2.33‐fold) diabetes risk; of profiles with two risk categories, the profile with family history and high lifestyle risk conferred the highest (4.18‐fold) diabetes risk; the profile with all three risk categories conferred 4.59‐fold increased diabetes risk.
- Associations between the risk category numbers and diabetes were more prominent in women and slightly more prominent in adults <55 years.
- Integrating comprehensive risk profiles is vital for effective prediction and prevention of diabetes, especially for women and younger adults.

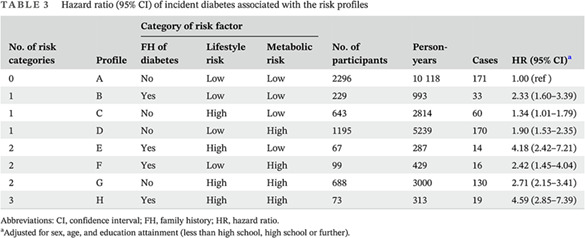

## 摘要

**背景:**

糖尿病家族史, 不健康的生活方式和代谢紊乱分别与较高的糖尿病风险相关, 但这三种风险类别的不同组合如何与糖尿病事件相关仍不清楚。我们旨在评估家族史, 生活方式以及代谢风险因素的综合风险概况与糖尿病风险的相关性。

**方法:**

这项研究包括5290名基线时无糖尿病的参与者, 平均随访时间为4.4年。对五种不健康的生活方式和五种代谢紊乱分别进行评分, 使生活方式和代谢风险综合评分从0到5不等。根据三个风险类别的组合构建了八个风险类别:糖尿病家族史(是, 否), 生活方式风险(高, 低)和代谢风险(高, 低)。

**结果:**

与无任何危险类别的相比, 随着危险类别的增加, 患糖尿病的风险逐渐增加:具有一个危险类别的风险比(HR, 95%可信区间[CI])为1.34(1.01~1.79)~2.33(1.60~3.39), 具有两个危险类别的为2.42(1.45~4.04)~4.18(2.42~7.21), 具有三个危险类别的HR为4.59(2.85~7.39)。风险类别的数量与糖尿病风险之间的关联在女性中更加显著(P_交互作用_=0.025), 在55岁以下的成年人中略显著(P_交互作用_=0.052)。

**结论:**

这项研究描述了全面的风险特征与糖尿病风险之间的关联, 在女性中观察到更强的关联, 在55岁以下的成年人中观察到略强的关联。

## INTRODUCTION

The increasing prevalence of diabetes has become a remarkable global public health concern. In 2021, 537 million adults suffered from diabetes globally, which is responsible for 6.7 million deaths.^1^ Notably, China has the highest number of adults with diabetes (approximately 140.9 million), accounting for 12.8% of adults in China.^1^, ^2^ Several risk factors, including family history (FH) of diabetes, unhealthy lifestyles, and metabolic disorders, have been identified as independent risk factors for diabetes.^3^, ^4^, ^5^ However, these heritable and environmental risk factors are implicated in the pathogenesis of diabetes with complex interplay and intricate gene–environment interactions,^6^ which increases the difficulty to predict diabetes risk by single or limited risk factors. Therefore, risk profiles encompassing multiple key factors would help identify subgroups at high risk of diabetes and contribute to diabetes prevention and control in countries like China that are encountering the epidemic of diabetes. Previous studies have explored the associations of unhealthy lifestyles and metabolic disorders with diabetes individually or collectively,^7^, ^8^, ^9^, ^10^, ^11^ but few studies have taken FH into account simultaneously when constructing risk profiles for diabetes. Given the considerable contribution of FH to the risk of diabetes,^12^ adding the FH of diabetes may improve the precision and effectiveness of risk profiles in the prediction of diabetes risk. Thus far, studies investigating diabetes‐related risk profiles comprising the FH of diabetes and lifestyle and metabolic risk factors are limited.

Therefore, in a prospective cohort study of Chinese adults, we constructed risk profiles based on the FH of diabetes, unhealthy lifestyles, and metabolic disorders and examined the associations between the risk profiles and diabetes risk, with particular interest in assessing whether the associations could be modified by sex and age groups.

## 1 METHODS

### 1.1 Study population

**FIGURE 1:**
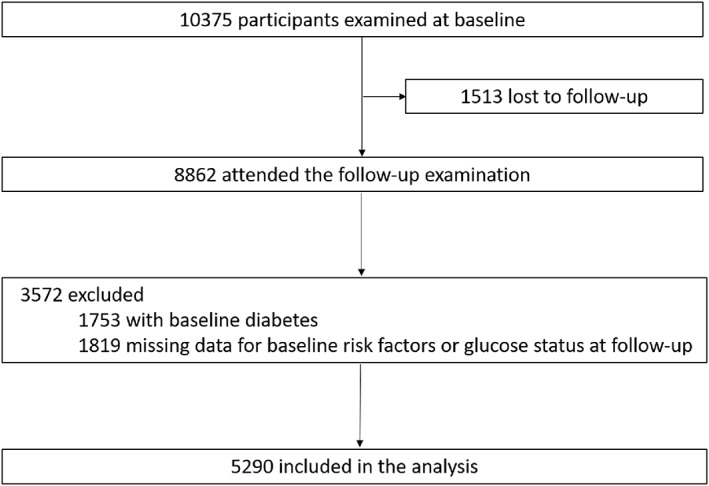
Flow chart of study participants.

The study participants were recruited from 10 communities in Jiading District, Shanghai, China.^13^ In brief, from March 2010 to August 2010, a total of 10 375 residents aged ≥40 years were recruited and received a survey including standard questionnaires, anthropometric measurements, and biochemical examinations. During August 2014 and May 2015, 8862 participants attended a follow‐up visit, of whom we excluded 1753 participants with diabetes at baseline and 1819 participants with missing data on baseline risk factors for diabetes or ascertainment of incident diabetes during the follow‐up. Finally, 5290 participants were yielded in the analysis (Figure 1). This study was approved by the Medical Ethics Committee of Ruijin Hospital, Shanghai Jiao Tong University. All study participants provided written informed consent.

### 1.2 Data collection

Trained technicians administered standardized questionnaires to collect data on demographic characteristics, FH, medication history, and lifestyle factors. The FH of diabetes was defined as having ≥1 first‐degree relative with diabetes. Education level was categorized as less than high school (<9 years) and high school or further (≥9 years). Sleep duration was divided into 6–8 h/day and <6 or >8 h/day. Sleep durations of <6 h/day and >8 h/day were combined into one category, because compared with sleep duration of 6–8 h/day, both shorter and longer sleep durations conferred increased diabetes risk in large prospective cohort studies.^4^, ^14^, ^15^ The leisure‐time physical activity was assessed by the International Physical Activity Questionnaire,^16^ and metabolic equivalent (MET) was calculated accordingly.^17^ Physical activity was classified as active (≥600 MET‐min per week) and inactive (<600 MET‐min per week). Average sedentary time was reported and classified into ≤4 h/day and >4 h/day. Heavy alcohol drinking was defined as >15 g/day for women and >30 g/day for men, consistent with robust evidence of healthy alcohol intake and incident diabetes.^10^, ^18^, ^19^

Waist circumference, height, and body weight were measured by trained physicians. Body mass index (BMI) was calculated as body weight (kg) divided by height in meters squared (m^2^). General obesity was defined as BMI ≥28 kg/m^2^. Abdominal obesity was defined as waist circumference ≥90 cm for men and ≥85 cm for women.^20^, ^21^ Three measurements of systolic and diastolic blood pressures obtained by an automated device (OMRON Model HEM‐752 FUZZY, Omron Co., Dalian, China) in a seated position after resting for ≥5 min were averaged for analysis. Hypertension was diagnosed as systolic blood pressure ≥140 mm Hg, diastolic blood pressure ≥90 mm Hg, or use of antihypertensive medications.

All participants underwent an oral glucose tolerance test (OGTT) after an overnight fast of ≥10 h, and blood samples were collected at 0 and 2 h during the test. Plasma glucose was measured on an autoanalyzer (Modular P800; Roche, Basel, Switzerland). Glycated hemoglobin A1c (HbA1c) was determined using the VARIANT II Hemoglobin Testing System (Bio‐Rad Laboratories, CA, USA). Insulin resistance was defined by a homoeostasis model assessment of insulin resistance (HOMA‐IR) in the highest sex‐specific quartile of the study participants, which was calculated by the formula: HOMA‐IR = fasting insulin (μIU/ml) × fasting glucose (mg/dl)/405.^22^ Serum concentrations of total cholesterol, triglycerides, high‐density lipoprotein (HDL) cholesterol, low‐density lipoprotein (LDL) cholesterol, fasting insulin, uric acid, and creatinine were measured with an autoanalyzer (Modular E170; Roche, Basel, Switzerland). Dyslipidemia was diagnosed as total cholesterol ≥6.22 mmol/L, triglycerides ≥2.26 mmol/L, HDL cholesterol <1.04 mmol/L, LDL cholesterol ≥4.14 mmol/L, or use of lipid‐lowering medications.^23^ A first‐voided early‐morning spot urine sample was collected for the measurement of urinary albumin on an autoanalyzer (Beijing Atom High‐Tech, Beijing, China), and the measurement of urinary creatinine on an autoanalyzer (Hitachi 7600–020, Tokyo, Japan). Urinary albumin‐creatinine ratio (ACR) was obtained by dividing urinary albumin concentrations (mg) by urinary creatinine concentrations (g). The estimated glomerular filtration rate (eGFR) was calculated from serum creatinine using the Chronic Kidney Disease Epidemiology Collaboration equation.^24^ Chronic kidney disease was defined as eGFR <60 ml/min/1.73 m^2^ or urinary ACR ≥30 mg/g.^25^ Hyperuricemia was defined as serum uric acid level ≥7.0 mg/dl (420 mmol/L) in men and ≥6.0 mg/dl (357 mmol/L) in women.

### 1.3 Construction of risk profiles

At baseline, we selected 11 risk factors based on the following criteria^1^: Sufficient evidence was available on their close associations with diabetes.^2^ They were relatively prevalent and of practical use in public health.^3^ Data on these risk factors were available in this cohort. Detailed selection rationales of 11 risk factors are shown in Data S1. We determined a lifestyle risk score based on five unhealthy lifestyles closely associated with diabetes risk: unhealthy sleep duration (<6 or >8 h/day),^4^, ^14^, ^15^, ^26^ physical inactivity (<600 MET‐min/week),^27^ sedentary behavior (>4 h/day),^28^ heavy alcohol drinking (>15 g/day for women or >30 g/day for men),^10^, ^18^, ^19^ and obesity (general obesity or abdominal obesity)^29^; a metabolic risk score based on five metabolic disorders of prominent diabetes risks: insulin resistance,^30^ dyslipidemia,^31^, ^32^, ^33^ hypertension,^5^, ^34^ chronic kidney disease,^35^ and hyperuricemia.^36^ We assigned one point (0 for absence and 1 for presence) for each risk factor. The lifestyle risk score ranged from 0 (the lowest risk) to 5 (the highest risk), and the lifestyle risk was categorized into low and high levels (low: 0–2; high: 3–5). The metabolic risk score ranged from 0 (the lowest risk) to 5 (the highest risk), and the metabolic risk was categorized into low and high levels (low: 0–1; high: 2–5). Eight risk profiles were constructed based on different combinations of three risk categories: without or with the FH of diabetes, low or high lifestyle risk, and low or high metabolic risk. We assigned letters to the eight profiles, with A referring to no risk categories (the lowest risk); B to D, one risk category; E to G, different combinations of two risk categories; and H, three risk categories (the highest risk).

### 1.4 Ascertainment of incident diabetes

Among participants without diabetes at baseline, incident diabetes during follow‐up visits was defined on the basis of the American Diabetes Association 2010 criteria: fasting plasma glucose ≥7.0 mmol/L, OGTT‐2 h plasma glucose ≥11.1 mmol/L, HbA1c ≥6.5%, or a self‐reported previous diagnosis of diabetes by healthcare providers.^37^

### 1.5 Statistical analysis

For baseline characteristics, continuous variables were expressed as means (SDs) and compared by one‐way analysis of variance, and categorical variables were expressed as numbers (proportions) and compared by chi‐square test.

Cox proportional hazards models were used to calculate hazard ratios (HRs) and 95% confidence intervals (CIs) for incident diabetes associated with individual risk factors and all risk profiles. In the time‐to‐event analysis, the censoring date for each participant was defined as the date of the diagnosis of diabetes, death, or the end of follow‐up, whichever came first, and the person‐time was from the enrollment date to the censoring date. To assess whether the associations between risk profiles and diabetes vary by sex and age, we repeated the main analyses by stratifications of sex and age groups (cutoff value: 55 years). Multiplicative interactions of risk profiles with sex and age group on incident diabetes were examined by including the product terms (eg, risk profile×sex) in the models. Statistical significance used a two‐sided *p* value of <.05. Analyses used SAS version 9.4.

## 2 RESULTS

Of 5290 study participants, the mean (SD) age was 57.4 (8.7) years, and 1878 (35.5%) were men (Table 1). Compared with participants without the FH of diabetes, participants with FH were younger, had higher educational attainment, were more likely to have a longer sedentary time, and had poorer metabolic profiles including higher levels of HOMA‐IR, fasting and OGTT‐2 h glucose, LDL cholesterol, total cholesterol, and eGFR. However, participants with FH had a lower proportion of hypertension and a lower level of systolic blood pressure than participants without FH. Compared with the participants excluded for missing data on baseline risk factor or ascertainment of incident diabetes during the follow‐up, the participants included in the analysis were more likely to be female and have lower educational attainment, and other baseline characteristics were similar (Table S1).

**TABLE 1:** Baseline characteristics of the study participants by FH of diabetes

Characteristic	Overall	FH of diabetes		*p* value
		Yes	No	
Number of participants	5290	468	4822	/
Age, years	57.4 (8.7)	55.3 (8.1)	57.6 (8.7)	<.001
Men, *n* (%)	1878 (35.5)	156 (33.3)	1722 (35.7)	.30
High school or further, *n* (%)	1043 (19.7)	134 (28.6)	909 (18.9)	<.001
Lifestyle factor				
Sleep duration, *n* (%)				
6–8 h/day	2366 (44.7)	215 (45.9)	2151 (44.6)	.58
<6 or >8 h/day	2924 (55.3)	253 (54.1)	2671 (55.4)	
Physical activity, *n* (%)				
<600 MET‐min/week	2177 (41.2)	199 (42.5)	1978 (41.0)	.53
≥600 MET‐min/week	3113 (58.9)	269 (57.5)	2844 (59.0)	
Sedentary time, *n* (%)				
≤4 h/day	2107 (39.8)	166 (35.5)	1941 (40.3)	.044
>4 h/day	3183 (60.2)	302 (64.5)	2881 (59.8)	
Alcohol drinking, *n* (%)				
Noncurrent heavy drinker	4883 (92.3)	427 (91.2)	4456 (92.4)	.36
Current heavy drinker	407 (7.7)	41 (8.8)	366 (7.6)	
Body shape, *n* (%)				
Nonobesity	3692 (69.8)	322 (68.8)	3370 (69.9)	.63
Obesity	1598 (30.2)	146 (31.2)	1452 (30.1)	
Metabolic factor				
Insulin resistance, *n* (%)	1323 (25.0)	134 (28.6)	1189 (24.7)	.058
HOMA‐IR	1.68 (1.08)	1.82 (1.22)	1.66 (1.07)	.006
Glucose profile				
Fasting glucose, mg/dl	5.1 (0.6)	5.2 (0.6)	5.1 (0.6)	.002
OGTT‐2 h glucose, mg/dl	6.6 (1.7)	6.9 (1.8)	6.6 (1.7)	.002
HbA1c, %	5.6 (0.3)	5.6 (0.4)	5.6 (0.3)	.008
Dyslipidemia, *n* (%)	1984 (37.5)	185 (39.5)	1799 (37.3)	.34
Lipid profile, mmol/L				
LDL cholesterol	3.18 (0.85)	3.29 (0.89)	3.17 (0.84)	.005
HDL cholesterol	1.34 (0.32)	1.32 (0.29)	1.35 (0.32)	.13
Triglycerides	1.60 (1.07)	1.70 (1.24)	1.59 (1.05)	.055
Total cholesterol	5.33 (0.97)	5.45 (1.03)	5.32 (0.97)	.006
Hypertension, *n* (%)	2644 (50.0)	190 (40.6)	2454 (50.9)	<.001
Blood pressure, mm Hg				
Systolic blood pressure	139.6 (19.4)	136.3 (19.0)	139.9 (19.4)	<.001
Diastolic blood pressure	82.7 (10.2)	82.3 (10.5)	82.8 (10.2)	.35
Chronic kidney disease, *n* (%)	370 (7.0)	32 (6.8)	338 (7.0)	.89
eGFR, ml/min/1.73 m^2^	90.5 (12.1)	91.8 (11.8)	90.4 (12.1)	.018
Hyperuricemia, *n* (%)	726 (13.7)	67 (14.3)	659 (13.7)	.70
Serum uric acid, mmol/L	293.9 (90.2)	299.3 (91.8)	293.4 (90.1)	.18

*Note:* Data are mean (SD) or *n* (%). Proportions might not sum to 100% due to rounding.

Abbreviations: eGFR, estimated glomerular filtration rate; FH, family history; HbA1c, hemoglobin A1c; HDL, high‐density lipoprotein; HOMA‐IR, homoeostasis model assessment for insulin resistance; LDL, low‐density lipoprotein; MET, metabolic equivalent; OGTT, oral glucose tolerance test.

**FIGURE 2:**
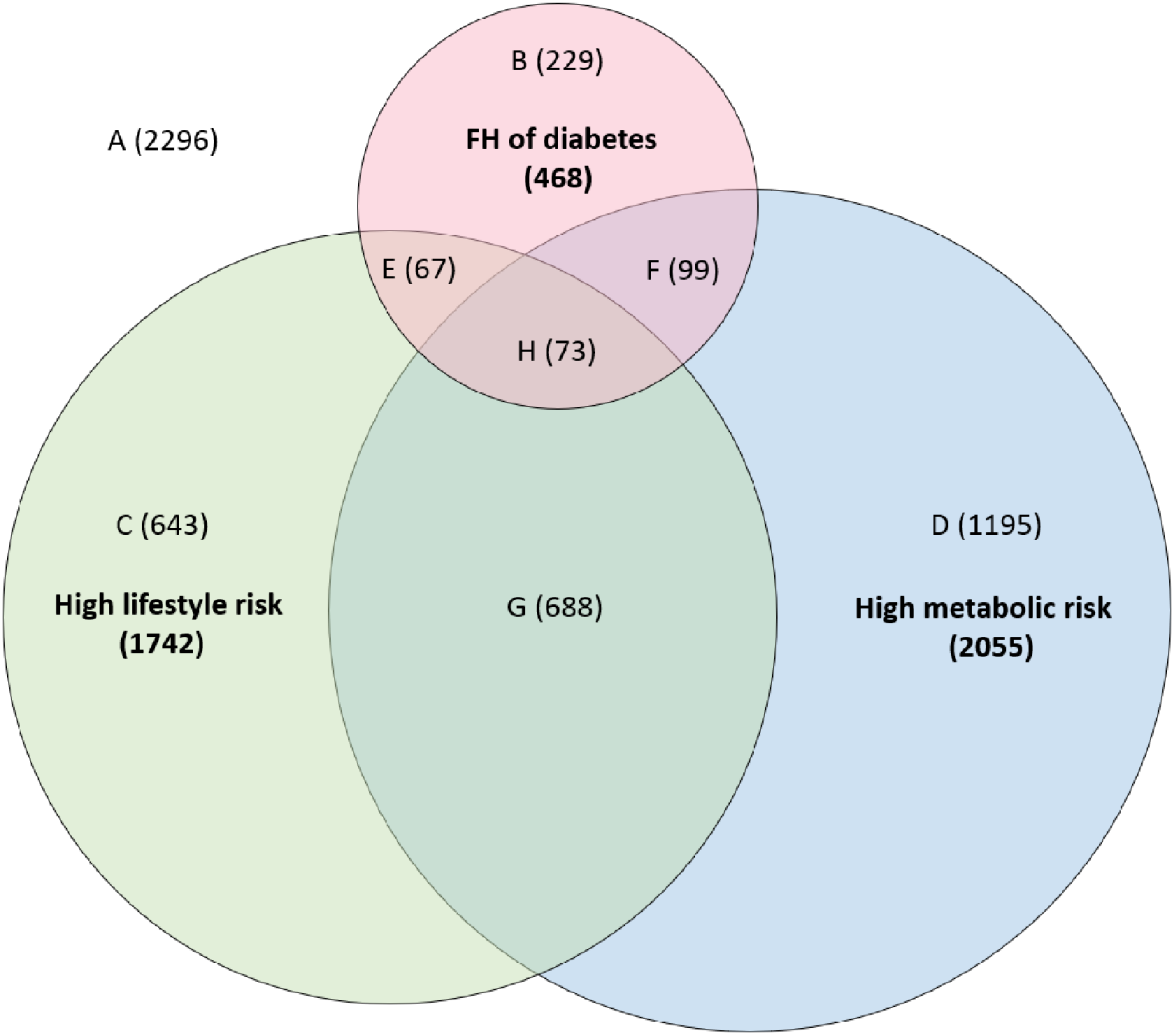
Venn diagram depicting the relationship between the risk profiles and the three risk categories among 5290 participants. The figure depicts eight risk profiles constructed based on different combinations of three risk categories: without or with the FH of diabetes, low or high lifestyle risk, and low or high metabolic risk. Letters A‐H were assigned to the eight profiles, with A referring to no risk categories (the lowest risk), B referring to only FH of diabetes, C referring to only high lifestyle risk, D referring to only high metabolic risk, E referring to the combination of FH of diabetes and high lifestyle risk, F referring to the combination of FH of diabetes and high metabolic risk, G referring to the combination of high lifestyle risk and high metabolic risk, and H referring to three risk categories (the highest risk). FH, family history.

Figure 2 depicts the distributions of eight risk profiles. Overall, 2296 participants without any risk category were categorized as profile A; 2067 participants with one risk category were categorized into profile B (only FH of diabetes, *n* = 229), profile C (only high lifestyle risk, *n* = 643), and profile D (only high metabolic risk, *n* = 1195); 854 participants with two risk categories were categorized into profile E (FH and high lifestyle risk, *n* = 67), profile F (FH and high metabolic risk, *n* = 99), and profile G (high lifestyle and metabolic risks, *n* = 688); 73 participants with all three risk categories were categorized as profile H.

During a mean follow‐up of 4.4 years (23 192 person‐years), 613 participants developed diabetes. When evaluating all 11 risk factors individually, FH was independently associated with a higher risk of diabetes; compared with lifestyle risk factors, metabolic risk factors showed stronger associations with diabetes in terms of number and effect size of risk factors that had a significant impact (Table 2). The number of incident diabetes was 171 (7.4%, 16.9 per 1000 person‐years), 33 (14.4%, 33.2 per 1000 person‐years), 60 (9.3%, 21.3 per 1000 person‐years), 170 (14.2%, 32.4 per 1000 person‐years), 14 (20.9%, 47.8 per 1000 person‐years), 16 (16.2%, 37.3 per 1000 person‐years), 130 (18.9%, 43.3 per 1000 person‐years), and 19 (26.0%, 60.7 per 1000 person‐years) among participants in profiles A to H, respectively (Table 3). Overall, participants with a greater number of risk categories exhibited higher risks of diabetes. Compared with profile A, profile H which included three risk categories exhibited the highest risk of diabetes (HR 4.59, 95% CI 2.85–7.39) of all other profiles. Of the three profiles with one risk category, profile B was associated with the highest risk of diabetes (HR 2.33, 95% CI 1.60–3.39), followed by profile D (HR 1.90, 95% CI 1.53–2.35) and profile C (HR 1.34, 95% CI 1.01–1.79). Of the three profiles with two risk categories, profile E was associated with the highest risk of diabetes (HR 4.18, 95% CI 2.42–7.21), followed by profile G (HR 2.71, 95% CI 2.15–3.41) and profile F (HR 2.42, 95% CI 1.45–4.04).

**TABLE 2:** Hazard ratio (95% CI) of incident diabetes associated with individual risk factors

Risk factor	HR (95% CI)	
	Model 1^a^	Model 2^b^
FH of diabetes	1.96 (1.55–2.48)	1.88 (1.49–2.38)
Lifestyle risk factor		
Unhealthy sleep duration	1.26 (1.07–1.49)	1.24 (1.05–1.46)
Physical inactivity	1.01 (0.86–1.19)	1.03 (0.87–1.21)
Sedentary behavior	1.14 (0.97–1.34)	1.10 (0.94–1.30)
Heavy alcohol drinking	1.09 (0.81–1.46)	1.12 (0.83–1.50)
Obesity	1.73 (1.47–2.03)	1.29 (1.08–1.54)
Metabolic risk factor		
Insulin resistance	2.15 (1.82–2.53)	1.70 (1.42–2.04)
Dyslipidemia	1.52 (1.30–1.78)	1.25 (1.06–1.47)
Hypertension	1.63 (1.37–1.94)	1.40 (1.17–1.67)
Chronic kidney disease	1.39 (1.07–1.82)	1.19 (0.91–1.55)
Hyperuricemia	1.44 (1.17–1.77)	1.07 (0.86–1.33)

Abbreviations: CI, confidence interval; FH, family history; HR, hazard ratio.

^a^ HRs (95% CIs) were adjusted for sex, age, and education attainment (less than high school, high school or further).

^b^ HRs (95% CIs) were further mutually adjusted for each individual risk factor on the basis of Model 1.

**TABLE 3:** Hazard ratio (95% CI) of incident diabetes associated with the risk profiles

No. of risk categories	Profile	Category of risk factor			No. of participants	Person‐years	Cases	HR (95% CI)^a^
		FH of diabetes	Lifestyle risk	Metabolic risk				
0	A	No	Low	Low	2296	10 118	171	1.00 (ref)
1	B	Yes	Low	Low	229	993	33	2.33 (1.60–3.39)
1	C	No	High	Low	643	2814	60	1.34 (1.01–1.79)
1	D	No	Low	High	1195	5239	170	1.90 (1.53–2.35)
2	E	Yes	High	Low	67	287	14	4.18 (2.42–7.21)
2	F	Yes	Low	High	99	429	16	2.42 (1.45–4.04)
2	G	No	High	High	688	3000	130	2.71 (2.15–3.41)
3	H	Yes	High	High	73	313	19	4.59 (2.85–7.39)

Abbreviations: CI, confidence interval; FH, family history; HR, hazard ratio.

^a^ Adjusted for sex, age, and education attainment (less than high school, high school or further).

**FIGURE 3:**
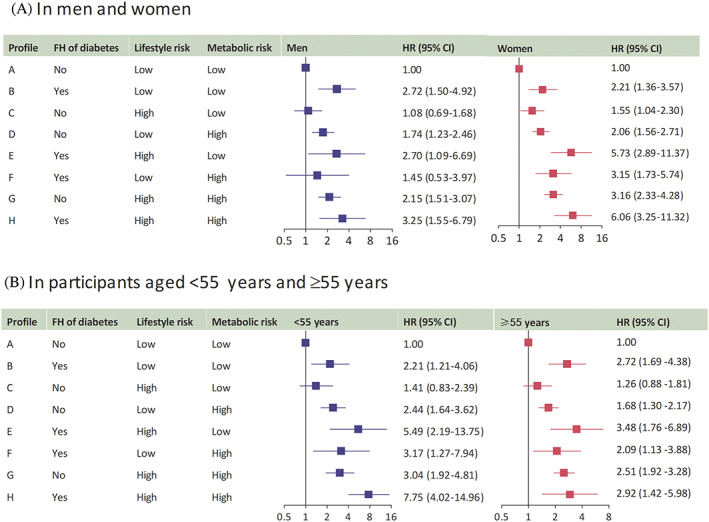
Hazard ratio (95% CI) of incident diabetes associated with the risk profiles by sex and age group. HRs (95% CIs) were adjusted for sex, age, and education attainment (less than high school, high school or further). CI, confidence interval; FH, family history; HR, hazard ratio.

Associations between the risk profiles and diabetes were substantially different between men and women (Figure 3A). Compared with profile A, the profiles with one risk category conferred comparable risks of diabetes between men and women: among men, the HRs (95% CIs) of diabetes associated with profiles B, C, and D were 2.72 (1.50–4.92), 1.08 (0.69–1.68), and 1.74 (1.23–2.46), respectively; among women, the corresponding HRs (95% CIs) were 2.21 (1.36–3.57), 1.55 (1.04–2.30), and 2.06 (1.56–2.71). The profiles with two risk categories exhibited higher risks of diabetes among women than men: among men, the HRs (95% CIs) for profiles E, F, and G were 2.70 (1.09–6.69), 1.45(0.53–3.97), and 2.15 (1.51–3.07), respectively; among women, the corresponding HRs (95% CIs) were 5.73 (2.89–11.37), 3.15 (1.73–5.74), and 3.16 (2.33–4.28). Similarly, the association between profile H with all three risk categories and diabetes was greater in women (HR 6.06; 95% CI 3.25–11.32) than in men (HR 3.25; 95% CI 1.55–6.79). The risk of diabetes was gradually increased with greater numbers of risk categories, and such associations were significantly amplified in women, indicating an interaction between sex and the number of risk categories on diabetes risk (*p*_interaction_ = .025; Table 4).

**TABLE 4:** Hazard ratio (95% CI) of incident diabetes associated with the number of risk categories by sex

No. of risk categories	No. of participants	Person‐years	Cases	HR (95% CI)^a^	*p* for interaction
Men (*n* = 1878)					.025
0	733	3257	72	1.00 (ref)	
1	754	3320	99	1.55 (1.14–2.11)	
2	358	1572	62	2.12 (1.51–2.98)	
3	33	142	8	3.25 (1.55–6.79)	
Women (*n* = 3412)					
0	1563	6861	99	1.00 (ref)	
1	1313	5726	164	1.95 (1.51–2.50)	
2	496	2144	98	3.30 (2.49–4.39)	
3	40	171	11	6.06 (3.25–11.31)	

Abbreviations: CI, confidence interval; HR, hazard ratio.

^a^ Adjusted for age and education attainment (less than high school, high school or further).

Associations between the risk profiles and diabetes were also varied by age (Figure 3B). Compared with profile A, the profiles with one risk category exhibited comparable risks of diabetes between the two age groups, whereas the profiles with two or all three risk categories were consistently associated with greater risks of diabetes among participants aged <55 years than among participants aged ≥55 years (Table 5). Among participants aged <55 years, the HRs (95% CIs) for profiles E, F, G, and H were 5.49 (2.19–13.75), 3.17 (1.27–7.94), 3.04 (1.92–4.81), and 7.75 (4.02–14.96), respectively; among participants aged ≥55 years, the corresponding HRs (95% CIs) were 3.48 (1.76–6.89), 2.09 (1.13–3.88), 2.51 (1.92–3.28), and 2.92 (1.42–5.98). The associations between the numbers of risk categories and diabetes were more evident among participants aged <55 years, with a borderline interaction between the number of risk profiles and age group on incident diabetes (*p*_interaction_ = .052).

**TABLE 5:** Hazard ratio (95% CI) of incident diabetes associated with the number of risk categories by age group categorized by 55 years

No. of risk categories	No. of participants	Person‐years	Cases	HR (95% CI)^a^	*p* for interaction
<55 years (*n* = 2152)					.052
0	1064	4666	54	1.00 (ref)	
1	781	3398	78	2.05 (1.44–2.90)	
2	272	1171	39	3.27 (2.15–4.96)	
3	35	149	11	7.82 (4.05–15.09)	
≥55 years (*n* = 3138)					
0	1232	5452	117	1.00 (ref)	
1	1286	5648	185	1.63 (1.29–2.05)	
2	582	2544	121	2.52 (1.95–3.25)	
3	38	164	8	2.92 (1.42–5.98)	

Abbreviations: CI, confidence interval; HR, hazard ratio.

^a^ Adjusted for sex, age, and education attainment (less than high school, high school or further).

## 3 DISCUSSION

To the best of our knowledge, this study is the first to delineate the associations of comprehensive risk profiles comprising the FH of diabetes, unhealthy lifestyles, and metabolic disorders with incident diabetes. In this prospective cohort of middle‐aged and elderly Chinese adults, compared with the profile without any risk category, for profiles with only one risk category, FH was associated with 2.33‐fold increased risk of diabetes, followed by high metabolic risk (1.90‐fold) and high lifestyle risk (1.34‐fold); for profiles with two risk categories, the combination of FH and high lifestyle risk was associated with 4.18‐fold increased risk of diabetes, followed by the combination of high lifestyle and metabolic risks (2.71‐fold) and the combination of FH and high metabolic risk (2.42‐fold); the profile with all three risk categories had 4.59‐fold increased risk of diabetes. Notably, associations between the numbers of risk categories and diabetes risk were more prominent in women and in adults younger than 55 years, suggesting a potential modification effect of sex and a modest modification effect of age.

In this study, among adults with only one risk category, those with FH exhibited the greatest risk of incident diabetes, independent from lifestyle and metabolic factors. Such independent and prominent impact of FH on diabetes was in line with the findings from the InterAct case‐cohort study, which demonstrated that FH remains a strong and independent risk factor for diabetes with the adjustment of various prominent risk factors of diabetes.^38^ Our study was partly in accordance with previous studies that lifestyle and metabolic risk factors, individually and collectively, were associated with diabetes.^8^, ^10^, ^11^, ^39^ The Nurses' Health Study and the Health Professionals Follow‐Up Study reported that various heathy lifestyle profiles comprising BMI, smoking status, physical activity, alcohol assumption, and diet were associated with extended gains in life lived without diabetes.^10^ The Cardiovascular Health Study yielded that of six clusters grouped according to 11 metabolic parameters; compared with the healthiest metabolic cluster, all the other five clusters exhibited significantly higher risks of diabetes.^8^ The China Cardiometabolic Disease and Cancer Cohort Study showed robust benefits of healthy lifestyles on the risk of diabetes regardless of metabolic status.^11^ Our study extended previous studies by incorporating FH of diabetes into the combination of lifestyle and metabolic risk profiles and providing new evidence of the association patterns of the three‐dimensional risk profiles with diabetes.

Interestingly, we found that compared with men, women exhibited greater risks of diabetes associated with the same number of risk categories. Our findings were partially consistent with data from the Nurses' Health Study and the Health Professionals Follow‐Up Study that women gained a longer life expectancy free of diabetes than men by adhering to a low‐risk lifestyle.^10^ The observed sex‐related disparities might be related to the diversities in biology, culture, lifestyle, environment, and socioeconomic status between men and women, although the exact mechanisms are not clear.^40^ We also observed a borderline significant interaction between age groups and the number of risk categories on diabetes. When possessing the same number of risk category, adults younger than 55 years were at higher risk of diabetes than their older counterparts. Similar age‐related decrease in diabetes risk associated with multiple risk factors such as obesity and dyslipidemia were observed in a recent study of 93 781 Chinese adults aged ≥40 years.^41^ Besides, a genome‐wide association study in the UK Biobank identified different genetic variations associated with age at diagnosis of diabetes, supporting the hypothesis that the pathogenesis of diabetes changes with age.^42^

This study has important clinical and public health implications. Taken into account both the independent effect and the coexisting status of these risk factors in the real world, we developed eight risk profiles comprising 11 risk factors including FH of diabetes, unhealthy lifestyles, and metabolic disorders and delineated the association patterns of the comprehensive risk profiles with incident diabetes. Importantly, unhealthy lifestyles and metabolic disorders are both modifiable risk factors for type 2 diabetes. In most cases, lifestyle risk factors could be modified by adopting healthy lifestyles, and metabolic disorders could be controlled directly by medication therapies. Our findings provide novel insights into the targeting risk profiles for individualized prevention and early intervention for diabetes.

Strengths of this study included reliable evaluations of risk factors including the FH of diabetes, unhealthy lifestyles, and metabolic disorders; the fully verified definition of incident diabetes; and the population‐based prospective design. Our study also has notable limitations. First, the discoveries of this study are based on a relatively short follow‐up duration. Although sufficient cases of incident diabetes (11.6%, 26.4 per 1000 person‐years) were documented, the sample size for specific subgroups was relatively small, and a longer follow‐up among a larger sample size could provide important long‐term association information between risk profiles and diabetes. Second, our study was conducted among Chinese adults aged 40 years or older; therefore, caution should be exercised when generalizing our findings to other ethnic or age groups. Third, information on diet was not available in this study, which may affect the comprehensiveness of lifestyle information and limit the understanding of diabetes risk associated with lifestyle. Fourth, although confounders have been cautiously controlled in the analyses, potential unmeasured confounding or reverse causality may not be fully excluded.

The present study assessed diabetes risk attributable to comprehensive risk profiles comprising the FH of diabetes, unhealthy lifestyles, and metabolic disorders, with stronger associations observed in women and slightly stronger associations observed in adults <55 years. Our findings highlight the importance of integrating comprehensive risk profiles for the effective prediction and prevention of diabetes, especially for women and younger adults.

## CONFLICT OF INTERESTS

None declared.

## Supporting information


**Data S1.** Rationale for selection of risk factors.
**Table S1.** Baseline characteristics of study participants included and excluded.^a^
