# Supplementary material for: Comprehensive risk profiles of family history and lifestyle and metabolic risk factors in relation to diabetes: A prospective cohort study
Source: J Diabetes. 2022 Jun 28;14(6):414–24. doi: 10.1111/1753-0407.13289 (PMC9366567; doi:10.1111/1753-0407.13289)
Supplement: Supplementary file 1 — Data S1. Rationale for selection of risk factors. Table S1. Baseline characteristics of study participants included and excluded.a [file JDB-14-414-s001.pdf]

Supplementary Material

Table of contents

**Supplementary Method 1.** Rationale for selection of risk factors	………………….2
**Supplementary Table 1.** Baseline characteristics of study participants included and excluded [a]	………………….6

Supplementary Method 1. Rationale for selection of risk factors

Risk factor	Epidemiological evidence	References
FH of diabetes	• In the U.S. population, FH of diabetes has a significant, independent, and graded association with the prevalence of diabetes. This association not only highlights the importance of shared genes and environment in diabetes but also opens the possibility of formally adding FH to public health strategies aimed at detecting and preventing the disease.	1
**Lifestyle risk factors**		
Unhealthy sleep	• For short duration of sleep (≤5-6 h/night), the relative risk of type 2 diabetes was 1.28 (95% CI 1.03-1.60); for long duration of sleep (>8-9 h/night), the relative risk was 1.48 (1.13-1.96), from a systematic review and meta-analysis of 10 studies with 107756 participants and 3586 incident cases of type 2 diabetes.<br>• Compared with 7-h/day sleep duration per day, the pooled relative risks for type 2 diabetes were 1.09 (95% CI 1.04-1.15) for each 1-h shorter sleep duration among individuals who slept <7 h per day and 1.14 (1.03-1.26) for each 1-h increment of sleep duration among individuals with longer sleep duration, from a meta-analysis of 10 prospective studies with 18443 incident cases of type 2 diabetes among 482502 participants with follow-up periods ranging from 2.5 to 16 years.<br>• Nearly half of all adults sleep either too little (commonly defined as ≤6 h sleep per day; up to 33% in the general population) or too much (commonly defined as ≥9 h sleep per day; up to 18% in the general population), from the National Health Interview Survey and a review of possible mechanisms underlying long sleep duration and type 2 diabetes.	2-5
Physical inactivity	• A risk reduction of 26% (95% CI 20%-31%) for type 2 diabetes was found among those who achieved 11.25 MET h/week (equivalent to 150 min/week of moderate activity) relative to inactive individuals. Achieving twice this amount of physical activity was associated with a risk reduction of 36% (95% CI 27%-46%), with further reductions at higher doses (60 MET h/week, risk reduction of 53%), from a systematic review and dose-response meta-analysis of	6

	prospective cohort studies.	
Sedentary behavior	• The adjusted HR (95% CI) of diabetes associated with sedentary behavior was 1.910 (1.642-2.222) based on meta-analysis results from 14 studies with a total of 26 700 participants.	7
Alcohol consumption	• Moderate alcohol intake (5-15 g/day for women and 5-30 g/day for men) has been consistently associated with diabetic benefits in large cohort studies.<br>• Based on 694 data sources of individual and population-level alcohol consumption, along with 592 prospective and retrospective studies on the risk of alcohol use, the risk of all-cause mortality, and of cancers specifically, rises with increasing levels of consumption, and the level of consumption that minimizes health loss is zero.	8-10
Obesity	• The adjusted HR (95% CI) of type 2 diabetes for participants in the highest quintile of baseline measures compared with those in the lowest was 4.3 (2.9-6.5) for BMI, 3.0 (2.0-4.3) for BMI at 50 years of age, 4.2 (2.8-6.4) for weight, 4.0 (2.6-6.0) for fat mass, 4.2 (2.8-6.2) for waist circumference, 2.4 (1.6-3.5) for waist-hip ratio, and 3.8 (2.6-5.5) for waist-height ratio, from the Cardiovascular Health Study.	11
**Metabolic risk factors**		
Insulin resistance	• High HOMA-IR was associated with an increased risk of diabetes (quartile 4 vs 1: HR 6.70, 95% CI 6.08-7.39), and the population-attributable risk for incident diabetes attributed to insulin resistance was approximately 24.4% (95% CI 23.6-25.2), from the China Cardiometabolic Disease and Cancer Cohort Study.	12
Dyslipidemia	• The combined effect of 23 HDL cholesterol-related single nucleotide polymorphisms on type 2 diabetes, as generated with use of the penalized robust inverse-variance weighted method (β 0.24, 95% CI 0.087-0.393, P-value=0.002) demonstrated that elevated LDL cholesterol levels significantly increased the risk of diabetes, from a two-sample Mendelian randomization.<br>• Abnormalities in triglycerides might be a fundamental factor in the pathogenesis of diabetes.<br>• Triglycerides, HDL cholesterol, and LDL cholesterol are risk factors in the development of type 2 diabetes, from reviews of the role and function of lipids in	13-15

	diabetes.	
Hypertension	• The multivariable adjusted HRs (95% CI) for incident diabetes across blood pressure categories of <120/75 mmHg, 120-129/75-84 mmHg, 130-139/85-89 mmHg and hypertension were 0.66 (0.55-0.80), 1.0 (referent), 1.45 (1.23-1.71), and 2.03 (1.77-2.32), respectively, from the Women's Health Study.<br>• The multivariable adjusted relative risks (95% CIs) for diabetes across blood pressure categories of 130-139/85-<89 mmHg and hypertension were 1.39 (1.14-1.69) and 1.76 (1.43-2.16), respectively, from the Osaka Health Survey.	16,17
Chronic kidney disease	• Compared to the fourth quintile of glomerular filtration rate, the odds ratios (95% CIs) of incident diabetes for the first, second, third and fifth quintiles were 2.32 (1.06-5.05), 1.76 (0.80-3.88), 1.26 (0.56-2.84) and 2.59 (1.18-5.65), respectively, from the Insulin Resistance Atherosclerosis Study.	18
Hyperuricemia	• The age- and sex-adjusted HRs (95% CIs) for diabetes were 1.30 (0.96-1.76) for the second, 1.63 (1.21-2.19) for the third, and 2.83 (2.13-3.76) for the fourth quartile of serum uric acid, in comparison with the first quartile. After adjustment for BMI, waist circumference, systolic and diastolic blood pressure, and HDL cholesterol, the HRs decreased to 1.08 (0.78-1.49), 1.12 (0.81-1.53), and 1.68 (1.22-2.30), respectively, from the Rotterdam Study.	19

Abbreviations: BMI=body mass index; CI=confidence interval; FH=family history; HDL=high-density lipoprotein; HOMA-IR=homeostasis model assessment of insulin resistance; HR=hazard ratio; LDL=low-density lipoprotein; MET=metabolic equivalent.

References:

1. Valdez R, Yoon PW, Liu T, Khoury MJ. Family history and prevalence of diabetes in the U.S. population: the 6-year results from the National Health and Nutrition Examination Survey (1999-2004). Diabetes Care 2007;30(10):2517-2522.
2. Cappuccio FP, D'Elia L, Strazzullo P, Miller MA. Quantity and quality of sleep and incidence of type 2 diabetes: a systematic review and meta-analysis. Diabetes Care 2010;33(2):414-420.
3. Shan Z, Ma H, Xie M, et al. Sleep duration and risk of type 2 diabetes: a meta-analysis of prospective studies. Diabetes Care 2015;38(3):529-537.
4. Luckhaupt SE, Tak S, Calvert GM. The prevalence of short sleep duration by industry and occupation in the National Health Interview Survey. Sleep 2010;33(2):149-159.
5. Tan X, Chapman CD, Cedernaes J, Benedict C. Association between long sleep duration and increased risk of obesity and type 2 diabetes: A review of possible

mechanisms. Sleep Med Rev 2018;40:127-134.
6. Smith AD, Crippa A, Woodcock J, Brage S. Physical activity and incident type 2 diabetes mellitus: a systematic review and dose-response meta-analysis of prospective cohort studies. Diabetologia 2016;59(12):2527-2545.
7. Biswas A, Oh PI, Faulkner GE, et al. Sedentary time and its association with risk for disease incidence, mortality, and hospitalization in adults: a systematic review and meta-analysis. Ann Intern Med 2015;162(2):123-132.
8. Li Y, Schoufour J, Wang DD, et al. Healthy lifestyle and life expectancy free of cancer, cardiovascular disease, and type 2 diabetes: prospective cohort study. BMJ 2020;368:l6669.
9. U.S. Department of Health and Human Services, U.S. Department of Agriculture. 2015–2020 Dietary Guidelines for Americans, 8th ed. 2015; https://health.gov/dietaryguidelines/2015/guidelines/ (accessed Dec 16, 2015)
10. GBD 2016 Alcohol Collaborators. Alcohol use and burden for 195 countries and territories, 1990-2016: a systematic analysis for the Global Burden of Disease Study 2016. Lancet 2018;392(10152):1015-1035.
11. Biggs ML, Mukamal KJ, Luchsinger JA, et al. Association between adiposity in midlife and older age and risk of diabetes in older adults. JAMA 2010;303(24):2504-2512.
12. Wang T, Lu J, Shi L, et al. Association of insulin resistance and β-cell dysfunction with incident diabetes among adults in China: a nationwide, population-based, prospective cohort study. Lancet Diabetes Endocrinol 2020;8(2):115-124.
13. Pan W, Sun W, Yang S, et al. LDL-C plays a causal role on T2DM: a Mendelian randomization analysis. Aging (Albany NY) 2020;12(3):2584-2594.
14. Berkowitz D. Gout, hyperlipidemia, and diabetes interrelationships. JAMA 1966;197(2):77-80.
15. Femlak M, Gluba-Brzózka A, Ciałkowska-Rysz A, Rysz J. The role and function of HDL in patients with diabetes mellitus and the related cardiovascular risk. Lipids Health Dis 2017;16(1):207.
16. Conen D, Ridker PM, Mora S, Buring JE, Glynn RJ. Blood pressure and risk of developing type 2 diabetes mellitus: the Women's Health Study. Eur Heart J 2007;28(23):2937-2943.
17. Hayashi T, Tsumura K, Suematsu C, Endo G, Fujii S, Okada K. High normal blood pressure, hypertension, and the risk of type 2 diabetes in Japanese men. The Osaka Health Survey. Diabetes Care 1999;22(10):1683-1687.
18. Eldin WS, Ragheb A, Klassen J, Shoker A. Evidence for increased risk of prediabetes in the uremic patient. Nephron Clin Pract 2008;108(1):c47-c55.
19. Dehghan A, van Hoek M, Sijbrands EJ, Hofman A, Witteman JC. High serum uric acid as a novel risk factor for type 2 diabetes. Diabetes Care 2008;31(2):361-362.

Supplementary Table 1. Baseline characteristics of study participants included and excluded [a]

Characteristic	Study participants	Excluded participants [b]
Number of participants	5290	3332
Age, year	57.4 (8.7)	58.7 (10.9)
Men, n (%)	1878 (35.5)	1349 (40.5)
High school or further, n (%)	1043 (19.7)	842 (25.3)
FH of diabetes, n (%)	468 (8.5)	304 (9.1)
**Lifestyle factor**		
Sleep duration, n (%)		
6-8 hours/day	2366 (44.7)	1590 (47.7)
<6 or >8 hours/day	2924 (55.3)	1742 (52.3)
Physical activity, n (%)		
<600 MET-min/week	2177 (41.2)	1335 (40.2)
≥600 MET-min/week	3113 (58.9)	1986 (59.8)
Sedentary time, n (%)		
≤4 hours/day	2107 (39.8)	1107 (33.4)
>4 hours/day	3183 (60.2)	2208 (66.6)
Alcohol drinking, n (%)		
Non-current heavy drinker	4883 (92.3)	3067 (92.0)
Current heavy drinker	407 (7.7)	265 (8.0)
Body shape, n (%)		
Non-obesity	3692 (69.8)	2278 (68.4)
Obesity	1598 (30.2)	1054 (31.6)
**Metabolic factor**		
Insulin resistance, n (%)	1323 (25.0)	833 (25.0)
HOMA-IR	1.68 (1.08)	1.89 (1.58)
Glucose profile		
Fasting glucose, mg/dL	5.1 (0.6)	5.2 (1.0)
OGTT-2h glucose, mg/dL	6.6 (1.7)	7.3 (3.1)
HbA1c, %	5.6 (0.3)	5.6 (0.6)
Dyslipidemia, n (%)	1984 (37.5)	1272 (38.2)
Lipid profile, mmol/L		
LDL cholesterol	3.18 (0.85)	3.15 (0.86)
HDL cholesterol	1.34 (0.32)	1.33 (0.31)
Triglycerides	1.60 (1.07)	1.61 (1.15)
Total cholesterol	5.33 (0.97)	5.28 (1.00)
Hypertension, n (%)	2644 (50.0)	1639 (49.2)
Blood pressure, mmHg		
Systolic blood pressure	139.6 (19.4)	139.2 (20.1)
Diastolic blood pressure	82.7 (10.2)	82.0 (10.3)
Chronic kidney disease, n (%)	370 (7.0)	268 (8.0)
eGFR, mL/min/1.73 m^2	90.5 (12.1)	90.7 (88.9)
Hyperuricemia, n (%)	726 (13.7)	545 (16.4)
Serum uric acid, mmol/L	293.9 (90.2)	302.9 (92.9)

Data are mean (SD) or n (%). Proportions might not sum to 100% due to rounding.

[a] 3332 participants were excluded due to missing data for baseline risk factors for diabetes or ascertainment of incident diabetes during the follow-up.

[b] There were 11 participants missing data for physical activity, 17 participants missing data for sedentary time, 15 participants missing data for OGTT-2h glucose, 4

participants missing data for triglycerides, 11 participants missing data for systolic blood pressure, and 9 participants missing data for diastolic blood pressure. Abbreviations: eGFR=estimated glomerular filtration rate; FH=family history; HbA1c=hemoglobin A1c; HDL=high-density lipoprotein; HOMA-IR=homoeostasis model assessment for insulin resistance; LDL=ligh-density lipoprotein; MET=metabolic equivalent; OGTT=oral glucose tolerance test.
